# CAREGIVERS’ PERCEPTIONS OF ADVANCE CARE PLANNING–RELATED DISCUSSIONS FOR PEOPLE LIVING WITH DEMENTIA

**DOI:** 10.1093/geroni/igae098.2582

**Published:** 2024-12-31

**Authors:** Yuchen Zhang, Susan Sereika, Jennifer Lingler, Jennifer Seaman, Corinne Pettigrew, Marilyn Albert

**Affiliations:** University of Pittsburgh School of Nursing, Pittsburgh, Pennsylvania, United States; University of Pittsburgh School of Nursing, Pittsburgh, Pennsylvania, United States; University of Pittsburgh School of Nursing, Pittsburgh, Pennsylvania, United States; University of Pittsburgh School of Nursing, Pittsburgh, Pennsylvania, United States; Johns Hopkins University School of Medicine, Baltimore, Maryland, United States; Johns Hopkins University School of Medicine, Baltimore, Maryland, United States

## Abstract

People living with dementia (PwD) experience progressive decline in functional abilities, including the execution of real-time medical decisions. Nevertheless, advance care planning (ACP) has the potential to mitigate healthcare overutilization and prevent ethical dilemmas at the end-of-life. We aimed to characterize caregivers’ understandings of ACP-related discussions between PwD and their healthcare clinicians. This secondary analysis leveraged data (n=431) from the cross-sectional, interviewer-administered survey, “Care Planning for Individuals with Dementia”, at Alzheimer’s Disease Centers nationwide. Statistical analysis utilized descriptive statistics and multinomial logistic regression. Surveyed caregivers had a mean age of 78.3 years, were predominantly white (88.5%), and were highly educated (mean=15.9 years). Most (95.1%) were the care recipient’s designated healthcare proxy and 30.9% of caregivers were aware of discussions between PwD and clinicians about medical treatment preferences. Caregivers (23.2%) who did not co-reside with PwD were 2.71 times more likely to report knowing such discussions occurred (p< 0.001). Caregivers who had higher dementia knowledge scores were more likely to indicate the need for further discussion between the proxy and clinicians regarding treatment preferences (OR=1.27, p=0.02); however, caregivers who endorsed knowing a lot about severe stage dementia were less inclined (OR=0.31, p=0.003). Lastly, caregivers of those in earlier stages were more likely to acknowledge the necessity for additional discussions about treatment preferences between PwD and clinicians (no impairment & MCI: OR=7.38, p=0.002; mild: OR=5.32, p=0.005). Overall, our results highlight the need for healthcare clinicians to conduct ACP-related discussions proactively and explicitly include likely proxies, especially during prodromal and earlier stages of dementia.

